# Graphene oxide liquid crystals: synthesis, phase transition, rheological property, and applications in optoelectronics and display

**DOI:** 10.1186/s11671-015-1139-1

**Published:** 2015-11-06

**Authors:** Feng Lin, Xin Tong, Yanan Wang, Jiming Bao, Zhiming M. Wang

**Affiliations:** Institute of Fundamental and Frontier Sciences, University of Electronic Science and Technology of China, Chengdu, 610054 People’s Republic of China; State Key Laboratory of Electronic Thin Films and Integrated Devices, University of Electronic Science and Technology of China, Chengdu, 610054 People’s Republic of China; Department of Electrical and Computer Engineering, University of Houston, Houston, TX 77204 USA

**Keywords:** Graphene oxide liquid crystal, Liquid crystal display, Electro-optical properties, Rheological properties

## Abstract

Graphene oxide (GO) liquid crystals (LCs) are macroscopically ordered GO flakes dispersed in water or polar organic solvents. Since the first report in 2011, GO LCs have attracted considerable attention for their basic properties and potential device applications. In this review, we summarize recent developments and present a comprehensive understanding of GO LCs via many aspects ranging from the exfoliation of GO flakes from graphite, to phases and phase transitions under various conditions, the orientational responses of GO under external magnetic and electric fields, and finally Kerr effect and display applications. The emphasis is placed on the unique and basic properties of GO and their ordered assembly. We will also discuss challenges and issues that need to be overcome in order to gain a more fundamental understanding and exploit full device potentials of GO LCs.

## Review

Graphene is an atomically thin carbon material in hexagonal structure and has drawn immense attention due to excellent electrical, thermal, mechanical, and chemical properties and potential device applications [1–5]. Graphene oxide (GO) is synthesized from graphite through wet chemical oxidation and subsequent exfoliation [6–11]. Since GO can be chemically reduced to graphene, initial interest in GO originated from the goal to produce graphene at low cost in large scale [12–15]. It was only after the full development of wet chemical exfoliation of GO that GO LCs were discovered, although LCs of graphene and carbon nanotubes were already observed in chlorosulfonic acid or sulfuric acid [16–18].

Xu and Gao were the first to report nematic phase and isotropic-nematic phase transitions of GO aqueous suspensions in 2011, followed by Kim et al. who investigated the influences of GO flake aspect ratio and NaCl ionic strength on phase transitions [7, 19]. After that, many more detailed studies of basic properties and potential device applications appeared. We believe it is time to review the rapid developments over the past few years and summarize what has been achieved and what the challenges are for future development. We will focus on the unique property of GO and point out how this basic property will affect the characteristic of GO LCs and related device applications. Both strengths and weaknesses of GO LCs will also be discussed.

The organization of this review is as follows: In “Synthesis of Graphene Oxide Liquid Crystals” section, we will talk about various methods that have developed to synthesize GO. In “Phase Properties” section, we will discuss the phase diagram, phase transitions, and their dependence on the mass/volume fraction, size/aspect ratio, salt concentration, and pH value. In “Rheological Properties” section, we will review orientational control and alignment under flow. Birefringence and orientational switch by magnetic and electrical field will be covered in “Magnetic/Electro-Optical Properties” section. We will summarize absorption and fluorescence, shape, and optical anisotropy and optical properties. In “Displays with GO LCs” section, we will discuss the optoelectronic applications. At last, we will conclude the review by conclusion and discussion of challenges and issues for future development.

### Synthesis of Graphene Oxide Liquid Crystals

Graphite oxide was first prepared by Brodie using KClO_3_ and HNO_3_ about 150 years ago [8]. This method was improved by Staudenmaier in 1898 and in 1937 by Hofmann who used concentrated H_2_SO_4_, HNO_3_, and KClO_3_ to produce highly oxidized graphite. However, this method was time-consuming (about 1 week) and hazardous because of the generation of toxic gases (ClO_2_ and NO_x_) [9, 10]. In 1958, Hummers reported a new method by replacing HNO_3_ and KClO_3_ with NaNO_3_ and KMnO_4_ [11]. Because the oxidation can be completed within 2 h below 45 °C, this Hummers method has been widely used especially after the first mechanical exfoliation of graphene in 2004. It was in the pursuit of producing graphene in large quantity using wet chemical exfoliation that liquid crystals of graphene and subsequently graphene oxide were discovered in 2010 and 2011, respectively [7, 16, 19–23]. Since then, much attention was attracted to the basic properties and new device applications of graphene-based liquid crystals.

#### Strategies for Graphite Oxidation

**Fig. 1:**
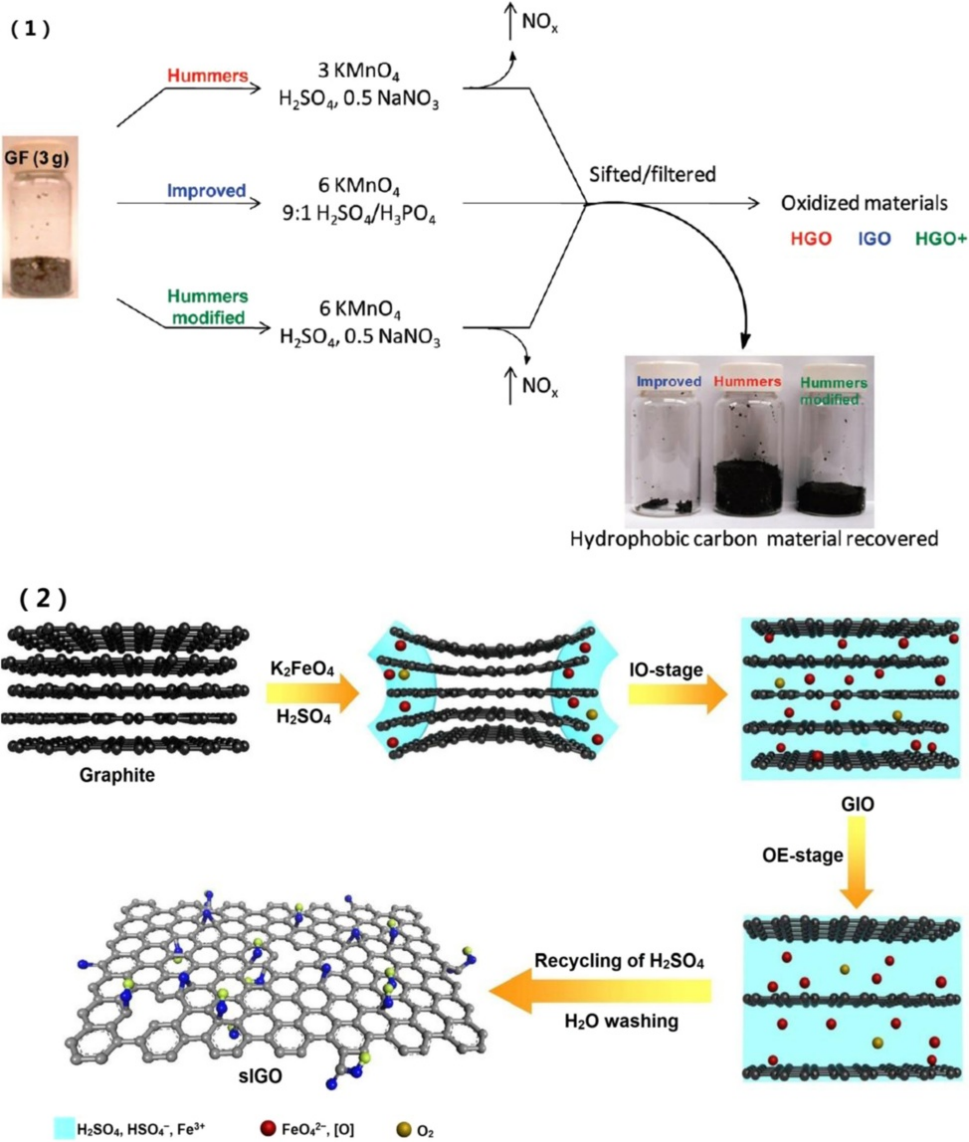
**1** Oxidation procedures of graphite flakes (GF) by Hummers method, improved Hummers method, and modified Hummers method. The *arrow* and nearby NO_x_ is gaseous nitric oxide. The *right side* shows generated graphite oxides as well as unoxidized or under oxidized graphite flakes that still exhibit hydrophobic property. Least amount of hydrophobic in the “improved” bottle indicates its highest oxidation efficiency [24]. **2** Synthesis of single layer graphene oxide (slGO) by K_2_FeO_4_. In intercalation oxidation (IO) stage, the in situ formed oxidants (FeO_4_^2−^ and atomic oxygen [O]) and O_2_ intercalate into graphite layers and form intercalated graphite oxide (GIO). Then, it is further oxidized and exfoliated by O_2_ in oxidation-exfoliation (OE) stage. With recycling of H_2_SO_4_ and water washing, slGO is obtained [25]

Hummers method has been modified to achieve high qualities of GO with large size or aspect ratio, high yield, and less toxic gases in short time[6]. Marcano and partners used a 9:1 mixture of concentrated H_2_SO_4_/H_3_PO_4_ and KMnO_4_ only to oxidize the graphite flakes with a higher reaction efficiency and less toxic gas [24]. Figure 1(1) shows GO of Hummers method (HGO), improved Hummers method (IGO), and modified Hummers method (HGO+) with additional KMnO_4_. It can be seen that IGO is more efficient than the other two methods.

To further increase the oxidation efficiency and reduce toxic chemicals, Peng and co-workers developed a green approach to producing GO without heavy metal and toxic gases [25]. As depicted in Fig. 1(2), when K_2_FeO_4_ is mixed with concentrated H_2_SO_4_, GO can be synthesized in 1 h at room temperature.

#### Exfoliation and Size Control of GO

After oxidation of graphite using the Hummers method, rapid heating and ultrasonic agitation are commonly used to exfoliate graphite oxide into a monolayer [6, 7, 21, 26]. However, these techniques always result in breakage of GO flakes into smaller pieces [6, 20, 27–30]. Aboutalebi and co-workers used large-sized graphite and pre-exfoliation process without sonication, creating ultra-large GO sheets with areas up to 10,000 μm^2^ and yield over 80 % [26, 31, 32]. Specifically, with graphite intercalation compounds prepared by stirring the mixture of graphite, H_2_SO_4_ and HNO_3_, then by thermal expansion at 1050 °C and oxidation with KMnO_4_ at room temperature, GO was obtained in deionized water by gentle hand shaking.

**Fig. 2:**
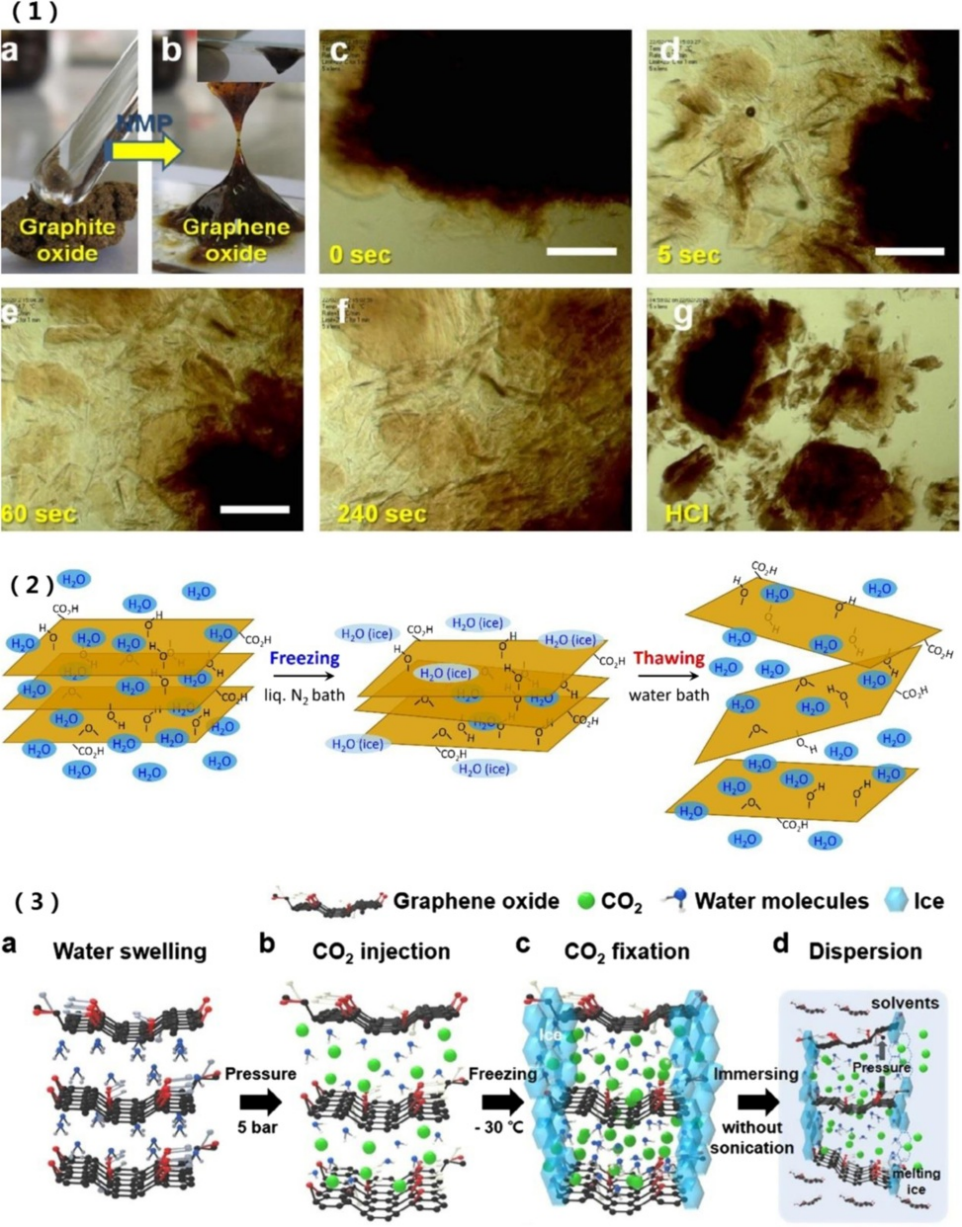
**1** Spontaneous exfoliation of graphite oxide in *N*-methyl-2-pyrrolidone (NMP) solvent. *a* Graphite oxide flakes. *b* GO gel-like dispersion in NMP. *c*–*f* Graphite oxide exposing to NMP and gradually exfoliates into thin flakes over time. *g* Graphite oxide in HCl solution. The scale bar: 100 μm [30]. **2** A sonication-free exfoliation method with a repetitive freeze-thaw cycle process [34]. **3** Sonication-free exfoliation of graphite oxide by interlayer CO_2_ injection. *a*, *b* graphite oxide swells in water and then high pressure CO_2_ is injected. *c* Cooling to −30 °C. *d* Immersed in water, the pressure of ejected CO_2_ gas separates GO layers [35]

With combinations of improvement in exfoliation and organic solvents, a modified strategy of spontaneous exfoliation of graphite oxide in polar aprotic solvents was proposed [30]. A class of organic solvents can be used to exfoliate graphite oxide by simple hand shaking without sonication; they include *N*-methyl-2-pyrrolidone (NMP), dimethyl formamide (DMF), dimethyl sulfoxide (DMSO), dimethyl acetamide (DMAc), and propylene carbonate (PC). Figure 2(1) shows the progress of exfoliation in NMP over time. A complete transformation from graphite oxide to GO is found in 240 s. In contrast, GO cannot be exfoliated in HCl [30]. From exfoliation study, it was also found that GO can be very well dispersed in various organic solvents such as DMF, *N*-cyclohexyl-2-pyrrolidone (CHP), tetrahydrofuran (THF), acetone, and ethanol [33].

When graphite oxide is dispersed in water, a sonication-free exfoliation method was proposed by Ogino et al [34]. This method is a repetitive freeze-thaw cycle which consists of fast freezing graphite oxide solution and then thawing of the frozen solid. As shown in Fig. 2(2), the graphite oxide aqueous solution is first freezing in a liquid N_2_ bath and then the sample with ice is thawing in the water bath. After six freeze-thaw cycles, graphite oxide is efficiently exfoliated with minimal fragmentation and yields about 80 % of GO. Further research finds that this method is effective for graphite oxide with high degrees of oxidation (C/O atomic ratios ≤2.6) for graphite structure retained would prevent the exfoliation. What is more, faster freezing rate is more efficient for exfoliation and forms high concentration GO dispersions [36]. Another sonication-free exfoliation method injects and maintains CO_2_ gas in interlayer of graphite interlayers by freezing and uses the ejection pressure of CO_2_ gas to disperse the graphite oxide [35]. In Fig. 2(3), graphite oxide swells in water with increasing the humidity and then high pressure CO_2_ is injected and cooled down to −30 °C to induce ice of water to surround the graphite oxide. As the surrounding ice melts, the pressure of ejecting gas spontaneously exfoliated and disperses the GO without sonication. The lateral size of GO exfoliated with above strategies is much larger than with sonication [26, 30, 35, 36]. Besides these sonication-free methods to maintain the lateral size of GO, a multi-step sonication exfoliation was studied without reducing the size of sheets but increasing the yield in contrast to continuous sonication [37].

Moreover, the optimized exfoliation method to achieve large size GO sheets, wide size, and shape distribution could be separated by size selection [38–40]. Some size separation methods based on density gradient ultracentrifuge [40] and pH selective precipitation [39] were put forward, but further purification process is needed for these methods. A facile spontaneous size selection technique without extra additives reveals that with proper concentration of GO dispersed in water, small size sheets and large size sheets will be separated, with small sheets forming isotropic phase and large sheet nematic phase for large size sheets [38].

Specified size of GO can be achieved by controlling starting graphite, oxidation, and exfoliation procedure. The large size of precursor natural graphite favors larger sheets though it is not necessary to form high lateral size GO [41, 42]. In the oxidation process, it is found that oxidation time, volume of oxidants, and oxidation path will also affect the GO particle size [41, 43–45]. Zhang et al. observed that the mean size of GO sheets descends with longer oxidation time and more oxidants [44]. It is also observed that higher oxidation degree with C/O of 2.08 shows larger GO size than it with C/O of 2.63 after same sonication [46]. With the increasing of oxidation degree, the content of oxygen-containing groups like hydroxyl and epoxide groups increased. The increasing oxygen-containing groups further increased the interlayer distance of graphite oxide and finally decreased the van der Waals interlayer interactions. As a result, the graphite oxide was easier to break into small pieces. Furthermore, the oxygen-containing groups decreased the bond energy between carbon atoms. Therefore, C-C bonds and graphite oxide cracked during ultrasonication. The oxidation paths mainly contain cross-planar and edge-to-center ways. The cross-planar oxidation results in periodic cracking of graphene sheets and reduces the lateral size [43]. If ultrasonication is adopted during exfoliation, the size of GO sheets decreases with the increase of sonication time [41, 44, 45]. It can be applied to reduce the GO particle size when small GO sheets are required in some application.

### Properties of GO LCs

#### Phase Properties

GO is the oxygenated form of graphene and GO dispersions exhibit stable nematic phase due to the electrostatic repulsive force which originates from hydrolysis of carboxyl and hydroxyl groups on the GO surface. The parameters which have critical influence on the phase transition of GO dispersions include mass/volume fraction [7, 19, 47], size/aspect ratio [7, 47, 48], salt concentration [49, 50], and the pH value [48, 49, 51] of solvents.

The phase transition of GO LCs from isotropic phase to nematic phase was firstly observed with the variation of volume/mass fraction by Xu et al. and Kim et al. [7, 19]. In their research, the influences of aspect ratio and NaCl on phase transition were also investigated. Then, Dan and partners observed the phase transition of giant graphene oxide (GGO) LCs [47]. Tkacz et al. and Zhao et al. reported the systematic research of phase transition based on pH value and salt almost at the same time [48, 51]. Recently, a range of arrested states (glass and gel) of GO dispersion were investigated with various volume fraction and salt concentration by Konkena et al. [50].

**Fig. 3:**
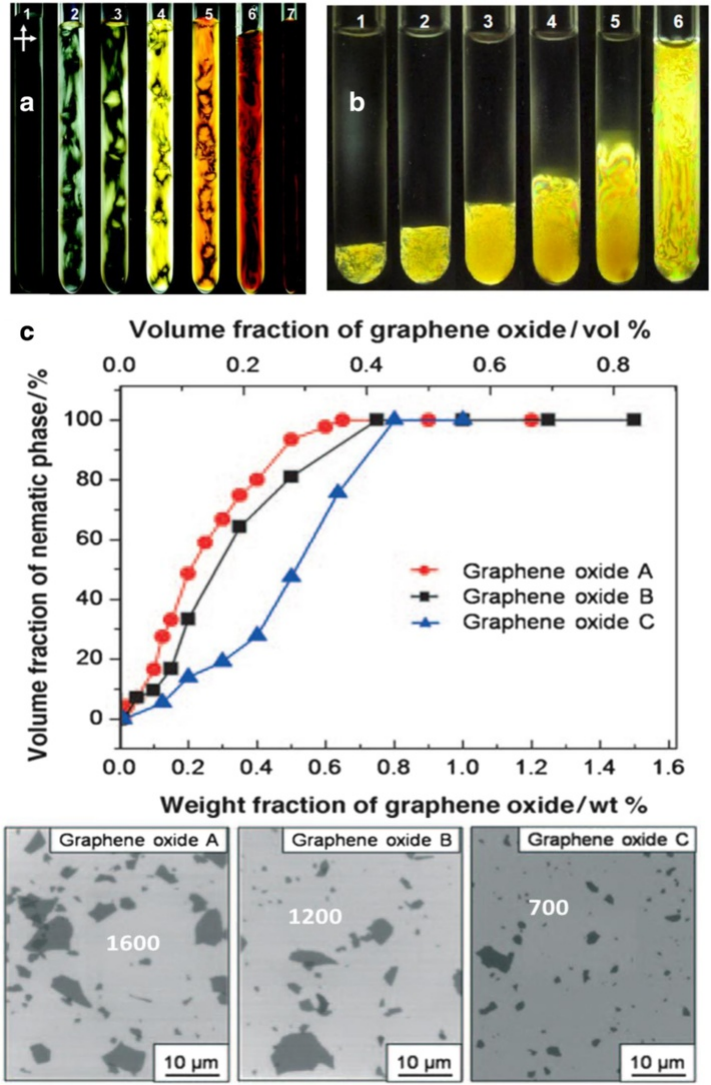
Birefringence images of GO dispersions with various mass fractions. **a** Birefringence images of GO dispersions in test tube with *f*_*m*_ 1 × 10^−4^, 2.5 × 10^−4^, 5 × 10^−4^, 1 × 10^−3^, 5 × 10^−3^, 1 × 10^−2^, and 2 × 10^−2^ (from 1 to 7) [19]. **b** Birefringence images of GO dispersions when nematic phase is separated from top isotropic phase after centrifuge and long-time standing. No relationship between labels in **a** and **b** [19]. **c** Nematic phase volume fraction versus graphene oxide concentration. Scanning electron microscopy (SEM) images of graphene oxide platelets exfoliated from various graphite sources with D/h aspect ratio of 1600, 1200, and 700, respectively [7]

The most obvious indication for a liquid crystal is the appearance of birefringence which can be observed between two cross-polarized optical filters. Figure 3a shows that birefringence begins to appear when the mass fraction (*f*_*m*_) of GO reaches 2.5 × 10^−4^, and the birefringence becomes stronger as the concentration of GO increases. It should be noted that these birefringent states are created dynamically by mixing and shaking the GO in water. The nature of equilibrium GO LC has to be determined after GO is completely settled. Due to large aspect ratio of GO, it takes several days for GO to settle down at the bottom of the tubes. Figure 3b shows typical separated and stable nematic phase of GO LCs after 4 h of centrifuge and long-time standing. The volume fraction of nematic phase increases with initial GO mass fraction. Figure 3c shows typical isotropic to nematic (I-N) phase transition, which is broad in general and varies with different GO sources [7]. The broad I-N transition is due to the large size polydispersity (83 %) of GO sheets [52], and the low transition point is due to large aspect ratio of GO [7, 19, 47]. Both observations agree with the Onsager theory model that large aspect ratio (about 2600) means lower phase transition point [53].

**Fig. 4:**
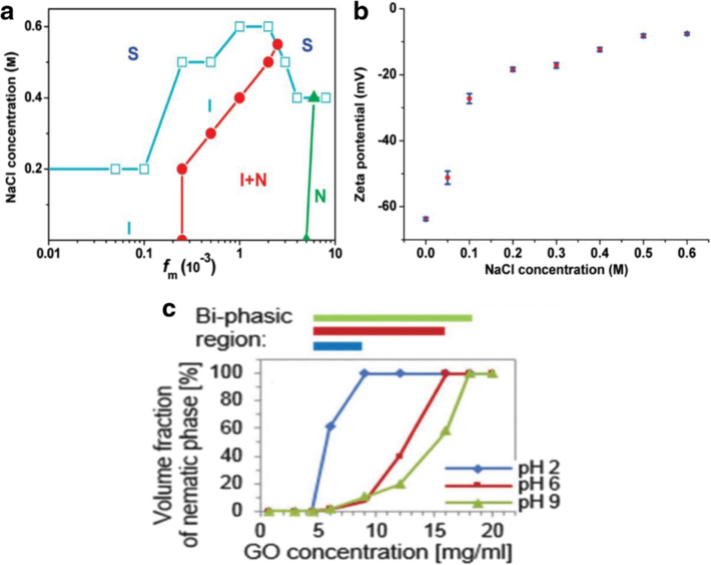
**a** Phase diagram of GO based on mass fraction (*f*_*m*_) and NaCl concentration. “I,” “I + N,” “N,” and “S” represent isotropic phase, isotropic-nematic state, nematic phase, and solid state of GO dispersions. **b** Zeta potential of GO dispersion (*f*_*m*_ = 0.005) versus NaCl concentration [19]. **c** Volume fraction of nematic phase as a function of GO concentration with pH value of 2, 6, and 9 [51]

As discussed before, GO is negatively charged due to carboxyl and hydroxyl groups on the surface. Like many other liquid crystals, GO LCs will be affected by solvent ion strength and pH level [19, 50]. The effect of phase of GO LC can be seen in Fig. 4a, when more of the NaCl is added, the biphasic GO LC can become isotropic and even collapse resulting in aggregated GO under a high NaCl level. This effect of salt can be understood from the reduced zeta potential shown in Fig. 4b. It is obvious that the repulsive force is dominant in GO dispersions and the dramatic decrease of GO interactions resulted in coagulation of dispersions. A high ion concentration screens the negative charge of GO flakes, leading to reduced electrostatic repulsive force between GO sheets [19, 50, 54].

Tkacz and co-workers investigated GO suspension in water with five different pH levels; the results are shown in Fig. 4c. The I-N transition can only be observed with pH values of 2, 6, and 9 [51]. The pH level will not affect the I-N transition point, but higher pH level will broaden the I-N transition region. This can be attributed to the influence of pH value on the ionization of surface functional groups. Increasing of the pH value improves the charge density of GO flakes and further promotes the fractionation of lateral size. As phase transition of GO dispersion is closely related with flake size/aspect ratio, increasing polydispersity will broaden the biphasic region. For extreme pH levels of 1 and 14, however, GO will form aggregation. In order to create stable GO LCs in solutions with high ionic strength and extreme pH level, Zhao and partners developed an amphiphilic polyelectrolytes with hydrophobic backbone and hydrophilic ionic groups (PHBIG) to absorb on GO sheets by hydrophobic forces from the water [48]. With this PHBIG to reduce the interfacial tension of GO sheets and water, GO LCs can be stably dispersed for relatively long time even in serum. This improvement in maintaining phase stability of GO LCs expands the application range to extreme conditions like electrolyte solutions and biological surroundings.

#### Rheological Properties

**Fig. 5:**
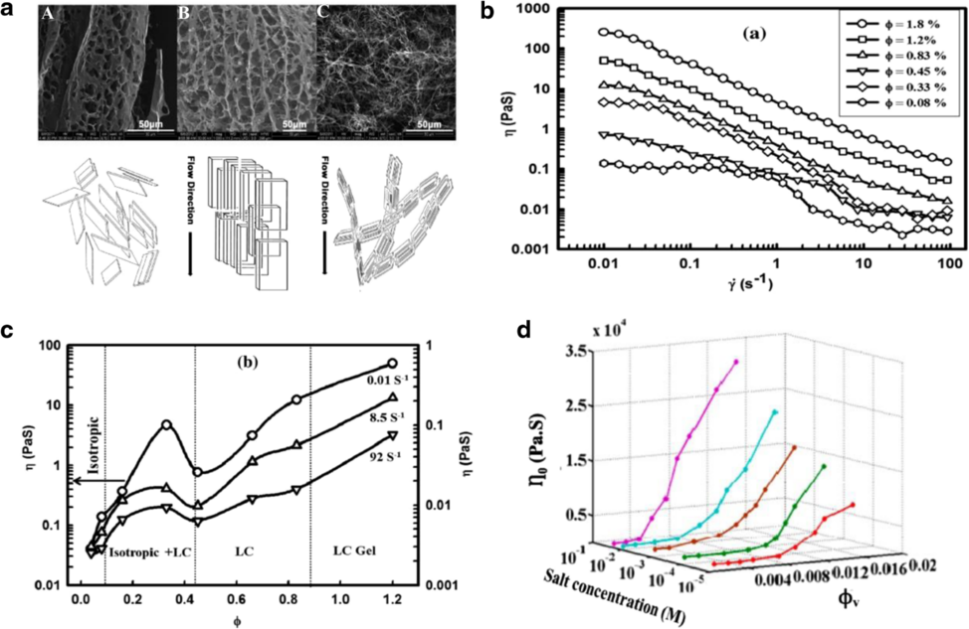
Rheological properties of GO dispersions. **a** Scanning electron microscope (SEM) images of GO dispersion under flow. *A* is the prepared GO dispersion with concentration of 5 mg/ml. *B* is the well-aligned structure under flow of the same GO dispersion. *C* is the flow-induced alignment of low concentration (1 mg/ml) of GO dispersion. *Bottom*: the corresponding flow-induced alignment schematics of GO dispersion [57]. **b** Relations between shear viscosity (*η*) and shear rate $\left(\dot{\upgamma}\right)$ with various volume fraction of GO dispersions. **c** The no-monotonic increasing of viscosity (*η*) with volume fraction (*ɸ*) [58]. **d** Zero shear viscosity (*η*_0_) of GO dispersions as a function of volume fraction (*ɸ*_*v*_) and salt concentration (*M*) [50]

Rheological properties played crucial roles in phase transition and device application of liquid crystals [55, 56]. Xu and Gao observed flow alignment from viscosity decrease during isotropic to nematic phase transition [19]. Then, Yang and partners investigated in detail with scanning electron microscope (SEM) [57]. Figure 5a shows the SEM images of flow alignment of GO dispersions with high and low concentration. The bottom of Fig. 5a shows the schematic of flow-induced alignment of GO LCs. Kumar et al. systematically studied the viscosity versus shear rate of GO suspensions with various shear rate and volume fractions [58]. As shown in Fig. 5b, the viscosity of GO suspensions decreases rapidly to a very small value with increasing of the shear rate. Specifically, GO dispersions exhibit Newtonian behavior in intermediate range at low concentration and typical shear thinning behavior at high concentration. This dramatic decrease of viscosity is resulted from shear alignment of GO sheets [19, 57]. Viscosity does not increase monotonically with GO concentration. As shown in Fig. 5c, at low concentration, the viscosity increases with GO concentration in the isotropic phase. It reaches the maximum and then decreases in the nematic phase with increasing of the volume fraction. The maximum point of viscosity is the transition point from isotropic to nematic phase.

Considering that the addition of salt would affect the phase transition of GO dispersions, Konkena et al. researched the viscosity of GO dispersions based on various salt concentrations [50]. Figure 5d shows the relations between zero shear viscosity and volume fraction as well as salt concentration. The viscosity of GO dispersions increases with volume fraction and salt concentration. At high salt concentration, viscosity increases very fast with the increasing of the volume fraction. However, GO dispersions show relatively small viscosity with low salt concentration while even if the volume fraction is pretty high. The addition of salt reduces the Debye screening length, screening the surface charges and enhancing the surface attractive force [59, 60], therefore, the viscosity goes up with the rising of salt concentration.

Besides viscosity analysis for shear flow and flow-induced alignment, the flow-induced GO flake ordering was measured with polarizing optical method and quantitatively analyzed by Hong et al [61, 62]. In their research, the motion model of GO flakes under flow was built and the corresponding order parameters were calculated.

#### Magnetic/Electro-Optical Properties

**Fig. 6:**
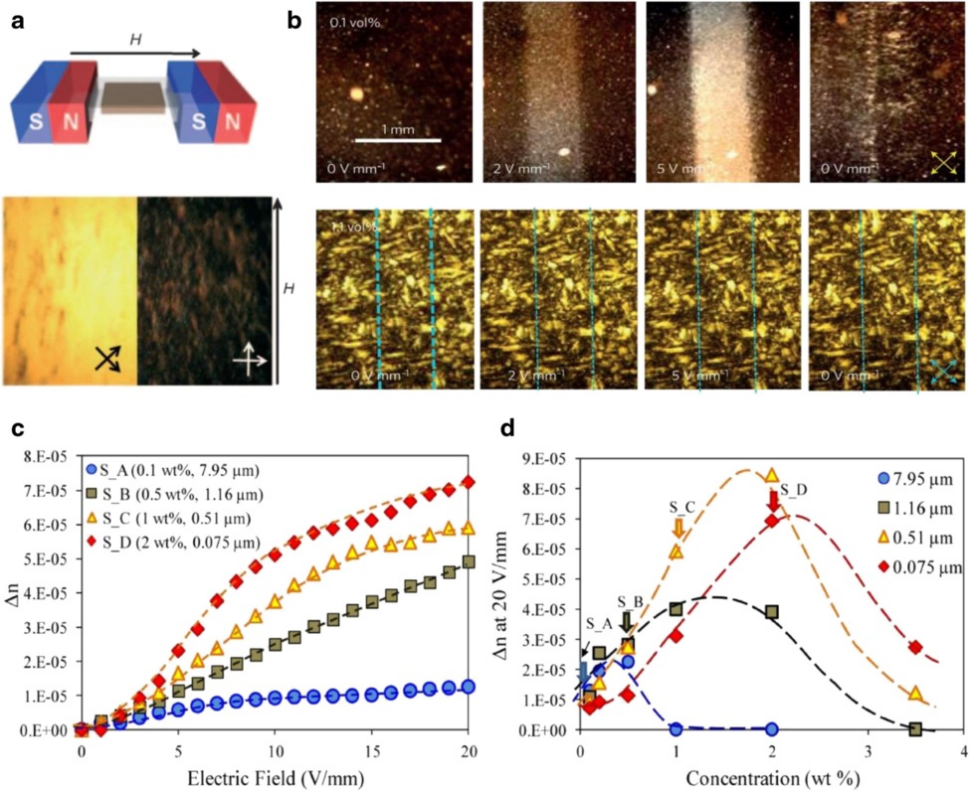
**a** Alignment of GO LCs with magnetic field. *Top*: experiment diagram of magnetic field-induced alignment. *Bottom*: texture of GO LCs aligned by magnetic field [7]. **b** Birefringence induced by electric field. *Top*: birefringence variation of 0.1 % GO dispersion under 10 kHz and various electric fields. *Bottom*: no birefringence change for 1.1 % GO dispersion with the same electric field [65]. **c**, **d** GO dispersion birefringence (Δ*n*) as a function of electric field (**c**) and as a function of concentration (**d**) for GO with different sizes under electric of 20 V/mm [66]

For potential device applications of GO LCs, it is important to be able to manipulate the orientation of GO flakes by external fields or forces [63, 64]. GO exhibits a weak magnetic susceptibility and can be aligned by a magnetic field. The top of Fig. 6a shows the experimental setup, and the birefringence pictures in the bottom figure indicate that the planes of GO flakes are aligned with the magnetic field. The problem is that the alignment process took about several hours because of the very weak magnetism of GO flakes. When GO was functionalized with magnetic iron oxide (Fe_2_O_3_), the alignment was completed in several seconds.

The alignment of GO by electric field is not straightforward because GO platelets will undergo electrophoretic migration and become electrochemically reduced under DC electric field [7]. Shen and co-workers solved this problem by employing high-frequency alternating current (AC) electric field [65]. The birefringence images in the top of Fig. 6b reveal the alignment of GO sheets with a 10-kHz electric field. The field of 5 Vmm^−1^ is about three orders of magnitude smaller than that for the switching of conventional molecular LCs. One serious problem with GO LCs is that the switching only works in low concentration (0.1 vol.%), not for LCs with higher GO concentration, as shown at the bottom of Fig. 6b.

Ahmad et al. investigated the effect of flake size, electric field, and concentration on field-induced birefringence [66]. Figure 6c shows that birefringence of all four samples increases with the electric field, and GO dispersions with smaller flake sizes exhibit higher birefringence; here, all four samples are in the I-N transition state. Figure 6d shows the dependence of birefringence on GO concentration under the electric field of 20 V/mm. It can be seen that there is an optimal concentration, which is also dependent on the GO flake size. GO LC with a mean size of 0.51 μm has the maximum birefringence at 2 % weight concentration.

The electric field-induced birefringence is a type of Kerr effect observed in nonlinear optical materials. The maximum Kerr coefficient of GO LCs obtained so far is 1.8 × 10^−5^ mV^−2^ [65] which is about three orders of magnitude larger than that of other optical materials [64, 67]. This high Kerr coefficient is a consequence of the large anisotropy of the polarizability of GO, relatively large flake spacing [68] as well as electrical double layer from surface oxygen functional groups [68–70]. As with the birefringence, the Kerr effect is dependent on the size and concentration of GO flakes.

**Fig. 7:**
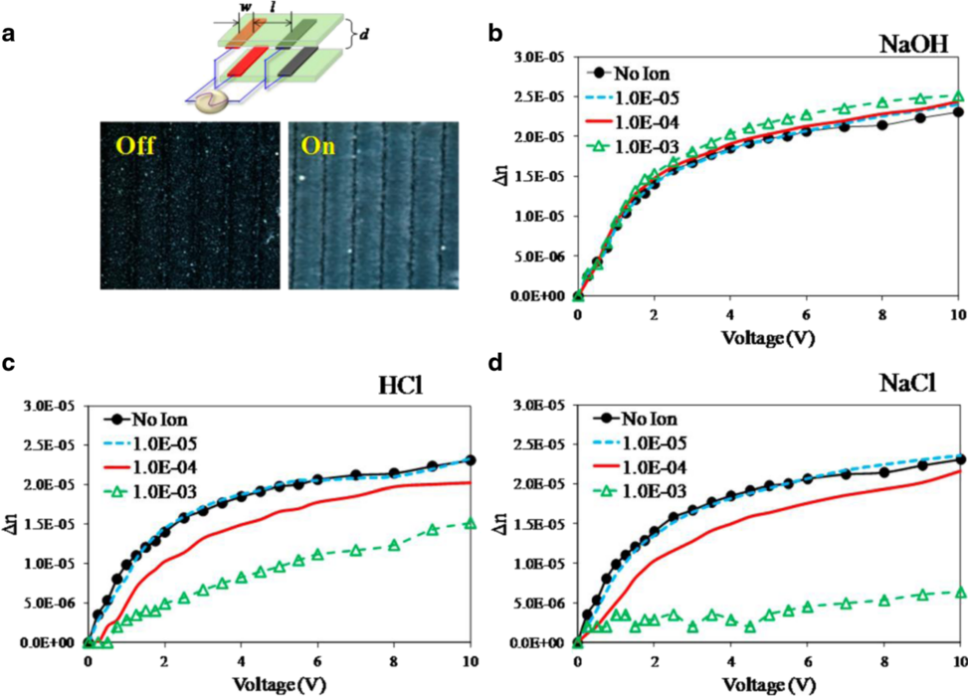
Electro-optical response based on different ionic solutions and concentrations. **a** The test cell model and images with and without electric field. **b**–**d** Dependence of birefringence (Δ*n*) on electric field and concentration for (**b**) NaOH-GO dispersion, (**c**) HCl-GO dispersion, and (**d**) NaCl-GO dispersion [49]

Because the ionic strength of solution will affect the interaction between GO flakes, it will certainly modify the birefringence behavior of GO LCs. Figure 7 shows that NaOH has a negligible effect; HCl and NaCl, especially NaCl, can reduce the birefringence by more than a half at a concentration of 10^−3^ M. This is probably because ionic solution is more effective in screening the external field. Therefore, it is important to reduce the concentration of residual salts of oxidation reagents during fabrication of GO dispersions for improved electro-optical performance of GO LCs [71].

#### Optical Absorption and Fluorescence Properties

**Fig. 8:**
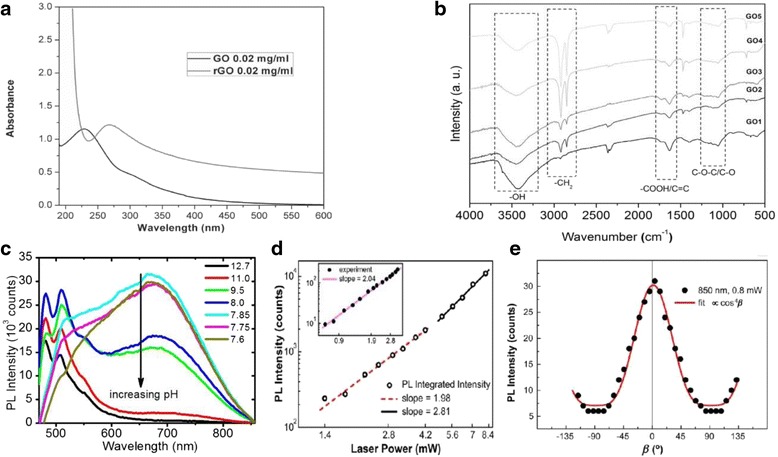
Optical absorption and fluorescence properties of GO. **a** UV-vis absorption spectra of GO dispersion and reduced GO (rGO) [26]. **b** Fourier transform infrared (FTIR) spectra of GO with different sizes. GO1, GO2, GO3, GO4, and GO5 have average size of 390, 200, 140, 60, and 38 nm [74]. **c** Photoluminescence (PL) spectra of GO dispersion with a different pH value [75]. **d** Fluorescence of GO as a function of laser power. **e** The PL intensity as a function of angle *β* between the plane of GO flake and polarization of excitation light [76]

Compared with conventional molecular LCs, GO LCs are very unique for strong optical absorption fluorescence in the UV and visible range. Figure 8a shows the absorption spectra of GO and reduced graphene oxide (rGO) [26]. The absorption spectrum of rGO is very similar to that of graphene, consisting of a broad feature extending to infrared wavelength and a UV peak at 275 nm due to π-π* transition from conjugated C-C bonds. In contrast, GO shows very weak absorption at wavelength longer than 500 nm, a shoulder near 300 nm due to n-π* transition from C = O bonds, and a blueshifted π-π* transition at 230 nm [24, 26, 30, 72, 73]. This big change in absorption from graphene to graphene oxide is a result of a change in the chemical composition and associated electronic band structure, namely, a decrease in conjugated C-C bonds and an increase in functional groups during oxidation and exfoliation. These functional groups such as C-O, -CH_2_, -OH, and -COOH can be observed with Fourier transform infrared (FTIR), as shown in Fig. 8b. The size-dependent spectra reveal that -CH_2_ is mainly formed on the edge of GO flakes, while other groups are attached to GO surfaces [74].

Unlike graphene, GO exhibits a broadband fluorescence ranging from UV, visible to near-infrared wavelengths [75–81], which opens up new device applications in optoelectronics and display [82]. The photoluminescence (PL) is believed to originate from electro-hole recombination in carbon clusters within carbon-oxygen matrix [79]. The PL of GO can be controlled by pH level [75]. As shown in Fig. 8c, the PL emission is centered around 500 nm in basic condition, but it redshifts to 680 nm in acidic condition. This spectral shift is due to electronic coupling of carboxylic acid groups to atoms of graphene backbone. It was also found that PL intensity was dependent on laser power and polarization of excitation laser [76]. As shown in Fig. 8d, the PL increases quadratically at low incident laser power and grows even faster at higher incident power. This nonlinear dependence of PL intensity can be attributed to multiphoton excitation [76]. The PL is also highly dependent on the angle *β* between the plane of GO flake and polarization of excitation laser, can be described as *I*_PL_∝cos^4^*β*. As shown in Fig. 8e, PL intensity reaches the minimum when polarization was perpendicular to the flake plane (*β* = ±90°) and the maximum PL is obtained when polarization was parallel to the flakes (*β* = 0°). This property can be used to identify the orientation of GO flakes in GO LCs [76].

### Displays with GO LCs

#### GO Back-Illuminated Liquid Crystal Display (LCD)

**Fig. 9:**
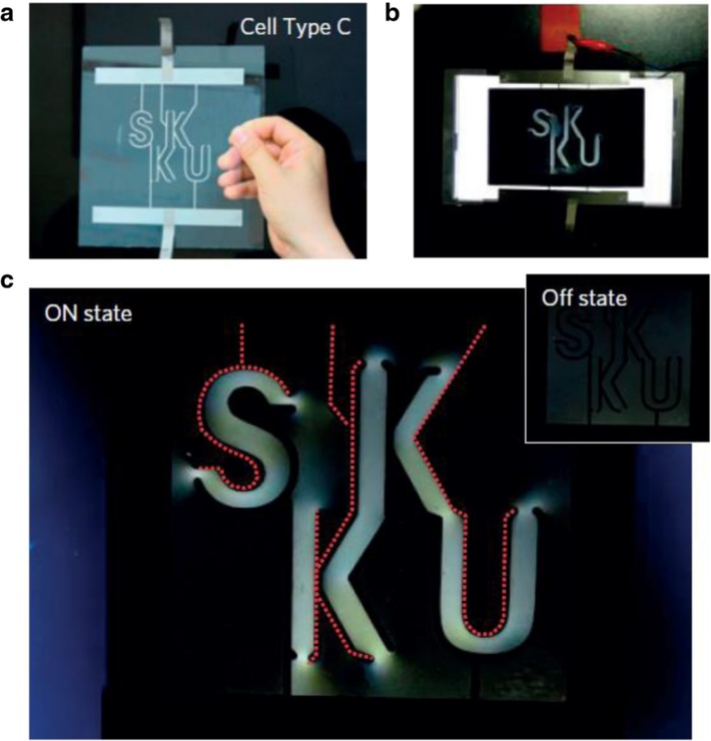
GO back-illuminated liquid crystal display (LCD) model. **a** Glass substrate with simple wire electrodes. **b** GO LCD on the *top* of back light. **c** Images of the device with electric field on and off [65]

Electric field-induced birefringence is the basis for many device applications of conventional liquid crystals. Similar applications, especially display, are also enabled by large Kerr effect of GO LCs [49, 65, 68]. A prototype of back light-illuminated GO liquid crystal display (LCD) is shown in Fig. 9, where the glass cell is filled with 0.056 vol.% GO LC, and the electric field is 20 V at 10 kHz. Compared with conventional LCDs, the device consumes low power and does not require special treatment for its electrodes [28, 41]. The size of the device, including the spacing between the top and bottom electrodes, can be significantly reduced. One problem with GO LCD is its slow on-off switching time, a few seconds for this device [68].

**Fig. 10:**
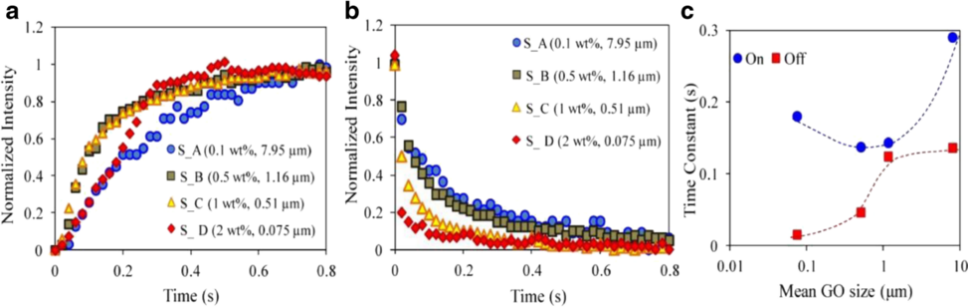
Dynamic response time of GO LCs with different flake sizes. **a**, **b** Rising and falling when electric field is turned on and off. **c** Dynamic response time constants for GO dispersions with various sizes [66]

A natural way to reduce the switching time is to reduce the size of GO flakes [66]. Figure 10a, b shows rising and falling responses of four GO LCs with sizes from ~10 μm down to sub 0.1 μm. The rising and falling time constants are summarized in Fig. 10c. It can be seen that both rising and falling time constants become shorter when the size decreases from 7.95 to 0.51 μm, but the rising time increases when the GO size is further reduced. This difference between rising and falling responses arises from different forces that govern the rotational dynamics of GO flakes. The rising time for the alignment of GO flakes is dependent on the anisotropy of polarizability and rotational viscosity. For very small GO flakes, polarization anisotropy decreases much faster than rotational viscosity, so the rising time increases. In contrast, the falling response is determined by rotational viscosity alone, smaller flakes suffer less from rotational viscosity, leading to reduced falling time. An optimal flake size is around 0.6 μm with average response time about one tenth of a second. Although this time is quite small, shorter switching time is required for certain applications such as TV and computer screens.

#### Rewritable and Reflective Displays

**Fig. 11:**
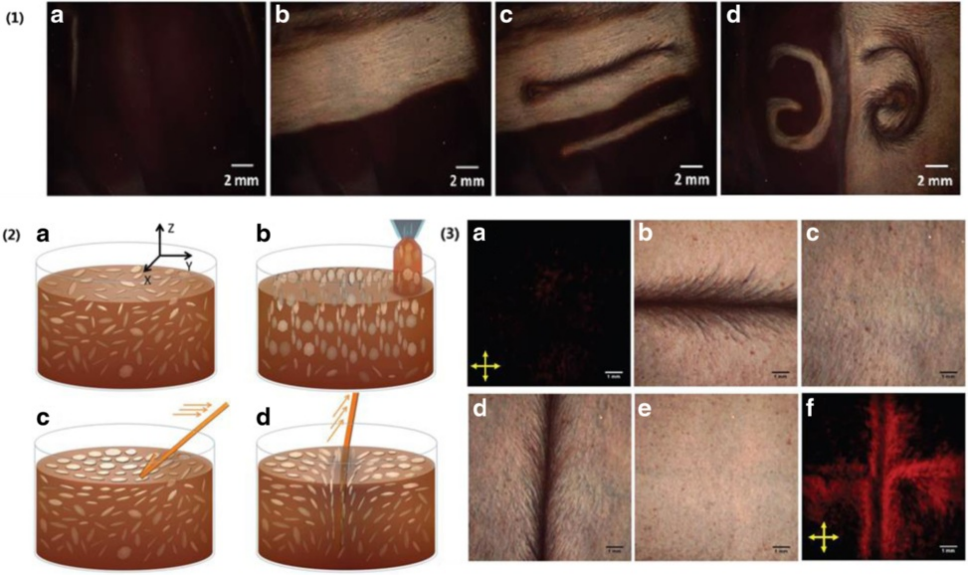
**1** Two display methods on the GO LCs surface. *a* A dark surface with weak light scattering. *b* A bright area created on a dark surface. *c* *Straight* and (*d*) *curved lines* created on dark and bright surface, respectively. **2** Orientation model of GO LCs during writing and erasing. *a* Isotropic phase of GO dispersions with random orientations. *Z* is perpendicular to the liquid surface. *b* Preparing of dark LC surface injecting GO dispersions with capillary tube onto a petri dish. The directors of GO flakes are parallel to *X*-*Y* surface. *c* Preparing of a bright surface by sliding a stick along the surface. The directors of GO flakes are perpendicular to LC surface. *d* Preparing of a *dark line* by sliding a stick horizontally in the LCs. **3** The rewritable and erasable properties of GO LCs. *a* Transmission image of isotropic GO LCs between two crossed optic polarizers. *b*–*e* Reflective images of GO LC surface with procedure of writing-erasing–writing-erasing. *f* Transmission image of GO LCs plotted in *e* [83]

In addition to LCD application using birefringence and back-illuminated light, the unique property of GO also enables a new type of display technology. Figure 11 demonstrates that GO LC can be used as a rewritable paper or board. Arbitrary features can be created and erased with a pen or stick [83]. Furthermore, the background of the LC surface can switch between dark as a black board and bright as a white board. This is an excellent example of reflective display that makes use of ambient light and does not need polarizing optics and back illuminating light. Due to low cost and energy consumption, reflective display has been widely used in electronic books such as Kindles. The reflective display in electronic books employ double-colored microcapsules that are black on half surface but white on the other half surface. The orientation of such “black-white pigment” can be controlled by electrical field. The reflective display technique used in Fig. 11 is very different: it makes use of unique property of GO flakes: strong optical anisotropy and absorption. For example, when planes of GO flakes are aligned with LC liquid surface, GO flakes will function as microscale mirrors, producing a shiny and white surface. When GO flakes are randomly oriented or with the planes in perpendicular to LC surface, they appear dark due to weak back scattering and strong absorption. As discussed above that the orientation of GO flakes can be controlled by electrical field, electrically controlled GO-based reflective display is expected in the near future.

## Conclusions

GO is unique compared to molecules in conventional LCs in that GO exhibits the largest diameter to thickness aspect ratio and largest shape and optical anisotropy. GO also shows magnetic response and can absorb and emit light in the visible and near-infrared range. These unique properties have made GO LCs very different from conventional ones in terms of basic phase property and device applications. Due to large aspect ratio and non-uniformity in size, stable GO LCs only show nematic phase and a broad phase transition from isotropic to nematic. Besides back-illuminated displays like conventional LCs, GO LCs can also be used in reflective display. The large Kerr effect and birefringence can be used in optoelectronic devices such as spatial phase modulators, Q-switch, saturable absorber, etc. The strong fluorescence allows GO to be used in many optical sensing and new type of display.

There are two big challenges. The first is to produce large quantity of GO with uniform size distribution. Uniform GO will enable new LC phases and will improve performance of GO devices. For example, it can help reduce the response time under electric field. The second challenge is the precise control and alignment of GO with high order parameter. Unlike rod-like molecules in conventional LCs, GO has more orientational degrees of freedom. An electric field cannot completely determine the orientation of a GO flake. A complete control of GO orientation and highly ordered alignment of GO will open up novel device applications.

## Abbreviations

(HGO+): GO of Hummers modified method
AC: alternating current
CHP: *N*-cyclohexyl-2-pyrrolidone
CNT: carbon nanotube
DMAc: dimethyl acetamide
DMF: dimethyl formamide
DMSO: dimethyl sulfoxide
FTIR: Fourier transform infrared
GF: graphite flakes
GGO: giant graphene oxide
GIO: intercalated graphite oxide
GO LCs: graphene oxide liquid crystals
HGO: GO of Hummers method
IGO: GO of improved method
I-N: isotropic to nematic
IO: intercalation oxidation
LCD: liquid crystal display
NMP: *N*-methyl-2-pyrrolidone
OE: oxidation-exfoliation
PC: propylene carbonate
PHBIG: polyelectrolytes with hydrophobic backbone and hydrophilic ionic groups
PL: photoluminescence
POM: polarized-light optical microscopy
rGO: reduced graphene oxide
SEM: scanning electron microscope
slGO: single layer graphene oxide
THF: tetrahydrofuran
UV: ultraviolet
